# Prostate carcinoma mimicking rectal cancer: a case report

**DOI:** 10.1093/jscr/rjae046

**Published:** 2024-08-29

**Authors:** Hajer Hassine, Sarra B Azouz, Habiba Debbabi, Dhouha Cherif, Haythem Yacoub, Beya Chelly, Hela Kchir, Nadia Maamouri

**Affiliations:** Gastroenterology B Department, Rabta Hospital, Tunis 2040, Tunisia; Gastroenterology B Department, Rabta Hospital, Tunis 2040, Tunisia; Gastroenterology B Department, Rabta Hospital, Tunis 2040, Tunisia; Gastroenterology B Department, Rabta Hospital, Tunis 2040, Tunisia; Gastroenterology B Department, Rabta Hospital, Tunis 2040, Tunisia; Gastroenterology B Department, Rabta Hospital, Tunis 2040, Tunisia; Gastroenterology B Department, Rabta Hospital, Tunis 2040, Tunisia; Gastroenterology B Department, Rabta Hospital, Tunis 2040, Tunisia

**Keywords:** prostate carcinoma, rectal cancer, differential diagnosis

## Abstract

In the pre-prostate specific antigen era, patients with prostate cancer (PC) commonly presented with symptoms. Currently, most PC are diagnosed at an asymptomatic stage with abnormal digital rectal examination or raised prostate specific antigen. In rare cases, PC may infiltrate the rectum and cause symptoms mimicking rectal cancer. It is difficult to differentiate between the two based on clinical features alone. We here report our experience in managing an 86-year-old male, with no significant personal pathological history, who presented with diarrhea and occasional rectal bleeding without any lower urinary tract symptoms. Investigations concluded to a PC invading the rectum and the patient was referred to urology department.

## Introduction

Prostate cancer (PC) was the second most common cancer in men worldwide in 2020 [1]. The most common presenting symptoms of PC are lower urinary tract symptoms including urinary complaints or retention, back pain, and hematuria. In rare cases, however, urinary symptoms may not be apparent, and patients can present with gastrointestinal symptoms instead. We present a case of PC invading the distal rectum and mimicking rectal adenocarcinoma. The work has been reported in line with the CARE guidelines.

## Case presentation

An 86-year-old male, with no significant personal or familial pathological history, presented with a few months diarrhea and occasional rectal bleeding without any lower urinary tract symptom. Physical examination was normal. Digital rectal examination detected a vegetant, friable hemi-circumferential mass without traces of blood on the gloved finger, situated at ~4 cm from the external anal orifice. Laboratory findings revealed no abnormalities.

**Figure 1:**
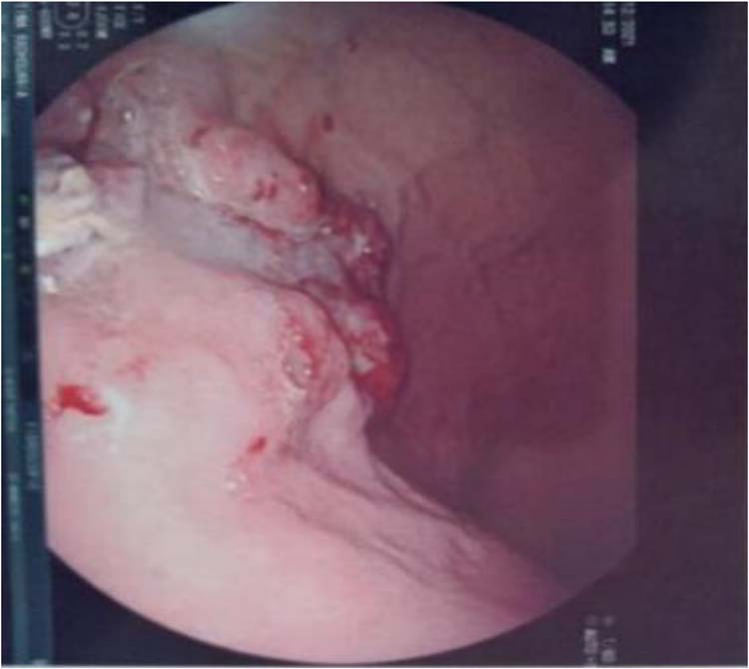
Large, circumferential, infiltrative, friable submucosal mass with erythematous overlying rectal epithelium, taking on the appearance of grape clusters.

**Figure 2:**
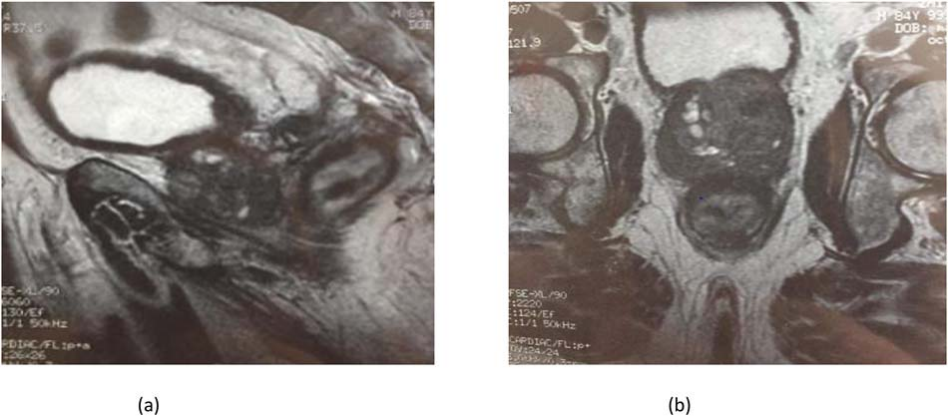
(a) MRI features: circumferential wall thickening narrowing the rectal lumen and invading the prostate and the mesorectum without extrinsic mass on sagittal section of the pelvis. (b) MRI features: a cross section of the pelvis showing circumferential wall thickening narrowing the rectal lumen and invading the prostate and the mesorectum.

**Figure 3:**
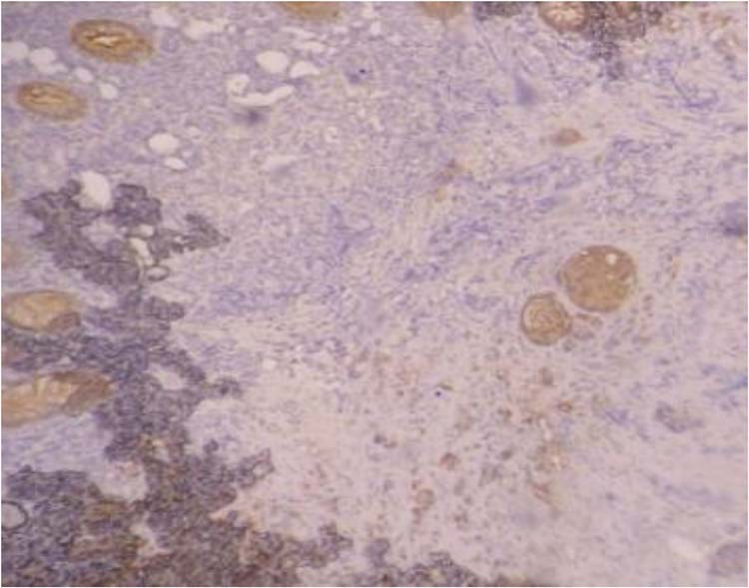
Positivity of immunohistochemical stains for pancytokeratin.

Serum carcinoembryonic antigen was 4.3 μg/L (normal value <5.1 μg/L). Biological tests showed normal platelet count and prothrombin time was 70%. Hemoglobin level was 12.2 g/dL. His biochemical tests showed hypoalbuminemia (31 g/dl). The patient was scheduled for a colonoscopy. However, a large bowel obstruction occurred during the bowel preparation. Thus, computed tomography (CT) scan of the abdomen and the pelvis was performed revealing circumferential wall thickening narrowing the rectal lumen and invading the prostate and the mesorectum with upstream bowel dilatation without extrinsic mass, lymphadenopathy, or metastasis. The patient underwent colostomy without incident. Colonoscopy was performed after colostomy, showing large, circumferential, infiltrative, friable, and stenosing submucosal mass with erythematous overlying rectal epithelium, taking on the appearance of grape clusters (shown in Fig. 1). Mucosal biopsies showed invasive poorly differentiated carcinoma referring at first a neuroendocrine tumor. Second mucosal biopsies were performed showing inflammatory modifications of the rectal mucosa without any malignancy. Third mucosal biopsies concluded to poorly differentiated carcinoma. Magnetic resonance imaging (MRI) of the pelvis showed circumferential wall thickening narrowing the rectal lumen and invading posterior peripheral area of the prostate, the bladder, the left seminal vesicle, and the mesorectum (shown in Fig. 2). The radiologist suggest a rectal cancer (RC) invading the urogenital tract or a PC invading the rectum subject to non-injected MRI. In fact, the MRI was interrupted before gadolinium injection because the patient was claustrophobic. Serum prostate specific antigen (PSA) was then ordered and it was raised at 25.59 μg/L (normal value<4 μg/L). Therefore, macro-biopsies of the rectal mucosa using snare loop were performed showing a poorly differentiated infiltrative carcinoma and immunohistochemical (IHC) stains were strongly positive for PSA and pancytokeratin and negative for anti-CD56 (shown in Fig. 3). Then, a clear diagnosis of PC invading the rectum was established. Bone scintigraphy was performed and was normal. The patient was referred to urology department. He underwent surgical castration. Then, androgen depletion therapy (ADT) was started. A total of 3 months later, there was a good response with his PSA level dropping to 0.5 μg/L. About 3 years after, patient is still alive, but PSA level has increased to 52 μg/L. The CT scan of the thorax, the abdomen, and the pelvis showed a prostatic hypertrophy with loss of the security edging with the rectum without any metastatic involvement.

## Discussion

Invasion of the rectum by PC is unusual, but some cases have been reported previously. The incidence of this uncommon condition was estimated to be between 1% and 11% [2]. It is rare because of the presence of the Denonvillier’s fascia, a thick capsule which separates the rectum from the prostate gland [3]. In the instance when PC does invade the rectum, it tends to insinuate between the two layers of Denonvilliers’ fascia, resulting in circumferential involvement around the rectum and ultimately fascial and mucosal penetration hence [4]. It can present with symptoms such as constipation, abdominal pain, rectal bleeding, and altered bowel habits, mimicking lower gut malignancies [5].

In our case, the patient experienced digestive symptoms without any lower urinary tract symptoms. Colonoscopy findings were uncommon showing a circumferential, infiltrative, friable, and stenosing submucosal mass, taking on the appearance of grape clusters. In historically reported cases, the diagnosis of PC was confirmed by histology obtained by endoscopic ultrasound with transrectal needle biopsy of the prostate or surgical biopsies [6]. In our case, PC invading the rectum was suspected after CT scan and MRI. Serum PSA was not requested in the beginning but seeing the radiological findings; it was ordered and was high suggesting a PC. Histology was not contributive initially at three times, what prompted us to perform macro-biopsies of rectal mucosa showing a poorly differentiated infiltrative carcinoma. The IHC stains were strongly positive for PSA.

The use of prostate-specific IHC stains in biopsies and serum PSA are useful in differentiating between the two. PSA is an important tool for early detection and differential diagnosis of PC, with a sensitivity and specificity of 92.30% and 40.81%, respectively [7]. In a case reported by Liu *et al.* [8], the pathologist performed prostate specific stains on the biopsies upon seeing a poorly differentiated, infiltrative carcinoma. They were positive for PSA and Alpha-methylacyl-CoA racemase (AMACR: a mitochondrial and peroxisomal enzyme overexpressed in PC). In fact, the diagnosis of PC invading the rectum is not always easy. In a review of the histopathological slides of 20 cases of PC found on colorectal biopsies by Lane *et al.* [9], it was found that it was difficult to differentiate between the two based on the histology itself. The authors recommended ruling out a prostatic origin when faced with a poorly differentiated carcinoma in a rectal biopsy with the use of prostate specific IHC stains.

In such cases, immunohistochemical and molecular diagnostic markers are very useful. Several studies evaluated the potential role of morphology and immunohistochemistry in resolving a primary origin of a poorly differentiated adenocarcinoma of the colorectal-prostatic region [10, 11]. On the aspect of prognosis, patients with PC invading the rectum usually have a short survival, which is often ˂30 months [7]. Our patient is still alive after a surveillance of ~26 months. He is in good general condition in relation to his age of 88 years. Our case is considered to be a castrate-resistant prostate cancer (CRPC) as PSA levels increased at follow up despite ADT. CRPC presents a spectrum of disease ranging from patients without metastases or symptoms with rising PSA levels despite ADT, to patients with metastases and significant debilitation due to cancer symptoms. Accordingly to recent guidelines, for rising PSA non metastatic CRPC like our case, ADT should be maintained. Patients with high risk non metastatic CRPC defined as PSA doubling time <10 months should be offered hormone therapy such as apalutamide. Men who are not considered high risk, secondary hormonal treatments may be attempted [12].

It’s important to perform colonoscopy for all patients presenting with lower digestive symptoms and to perform per-endoscopic biopsies if macroscopic lesion is present. Rectal biopsies confirm the diagnosis of rectal adenocarcinoma and eliminate other diagnoses. PC is diagnosed on the basis of MRI or prostate biopsy.

## Conclusion

In rare instances, PC can infiltrate the rectum and present with symptoms mimicking RC even without lower urinary tract symptoms. It is difficult to differentiate between the two based on clinical features alone. A high index of suspicion is required to avoid it being missed, delayed, or misdiagnosed especially since the patient may not have any prior history of PC, urinary symptoms, or PSA screening tests before.

## Conflict of interest statement

None declared.

## Funding

None declared.

## Patient consent

Patient gave an informed consent.
